# Complete Genome Sequence of a *Myoviridae* Bacteriophage Infecting *Salmonella enterica* Serovar Typhimurium

**DOI:** 10.1128/genomeA.00940-16

**Published:** 2016-09-29

**Authors:** Rubina Paradiso, Massimiliano Orsini, Sergio Bolletti Censi, Giorgia Borriello, Giorgio Galiero

**Affiliations:** a Istituto Zooprofilattico Sperimentale del Mezzogiorno, Portici, Italy; b Istituto Zooprofilattico Sperimentale G. Caporale, Teramo, Italy; c Cosvitec s.c.ar.l., Naples, Italy

## Abstract

The bacteriophage 118970_sal3 was isolated from water buffalo feces in southern Italy, exhibiting lytic activity against *Salmonella enterica* serovar Typhimurium. This bacteriophage belongs to the *Myoviridae* family and has a 39,464-bp double-stranded DNA (ds-DNA) genome containing 53 coding sequences (CDSs).

## GENOME ANNOUNCEMENT

Salmonellosis is one of the most frequent foodborne zoonoses representing a worldwide major public health concern. The main sources of infection for humans include meat products, eggs, vegetables, and water (1). Genomic diversity of *Salmonella* is increasingly studied, but at the same time, we have limited knowledge of *Salmonella* phage diversity. In this study, we isolated and characterized the bacteriophage 118970_sal3 infecting *Salmonella enterica* serovar Typhimurium. The phage was isolated from water buffalo feces, as in this animal species, this pathogen is a leading cause of diarrhea (2). Phage DNA was purified with the QIAamp DNA minikit, as described by the manufacturer. DNA sequencing was performed using the Ion Torrent PGM platform, yielding a total of 335,919 reads with an average read length of 239 bp and an average coverage of 2,034×. Quality control and trimming were carried out using in-house-implemented Python scripts, assembly was performed using the SPAdes software (3), and finishing was completed using the DraftDoctor software (version 1.0 CRS4; M. Orsini, unpublished data). Genome annotation was manually curated after a preliminary annotation performed by Prokka (4).

The 118970_sal3 genome consisted of 39,464 bp, with a G+C content of 50.7%. The genome contained 53 predicted CDSs. Of those CDSs, 29 matched identified phage genes, including 11 genes involved in phage structure, six genes involved in DNA replication, four genes involved in phage physiology, four genes involved in the phage lysis process, and four regulatory genes. Three genes encoded phage-related putative proteins, while the remaining 21 CDSs encoded hypothetical proteins. No genes associated with antibiotic resistance were identified. Further analysis, however, will be required to assign potential functions to the several unidentified and hypothetical genes.

Blast analysis indicated that the bacteriophage 118970_sal3 belongs to the *Myoviridae* family and showed 86.1% identity (determined with EMBOSS stretcher) to *Salmonella* Typhimurium phage ST64B (5), thus suggesting a genomic architecture similar to that of phage λ.

Phage diversity has a profound impact on environment, ecology, and prokaryotic evolution. Moreover, genomic characterization of different *Salmonella* phages may provide insights into the evolution of this pathogen, spread of virulence genes, and antibiotic resistance within this bacterial genus.

### Accession number(s).

The complete genome of the bacteriophage 118970_sal3 has been deposited in GenBank under the accession number no. KU927493.
